# Rural Residents’ Hopes and Fears about Aging in Place: The Need to Improve Access to Aging Resources

**DOI:** 10.1093/geroni/igab046.2363

**Published:** 2021-12-17

**Authors:** Allyson Brothers, Yuquin Jiao, Sue Schneider, Kaitlyn Wright

**Affiliations:** Colorado State University, Fort Collins, Colorado, United States

## Abstract

To support older adults’ preferences to age in place, home and community-based aging-related resources are available, but are often under-utilized. Many barriers prevent individuals from accessing aging-related resources, especially in rural and geographically isolated locations. Therefore, we set out to better understand the perspectives of community members who plan to age in place in rural areas. We administered a survey as part of a broader university-community partnership called Senior Access Points (SAP), which addresses aging-related resource access. Participants were N = 210 individuals living in rural regions across Northern Colorado, ranging from 37 to 94 years old (mean age = 68.91, SD = 8.85). We assessed hopes and worries about growing older at home, and awareness of available resources. Two independent coders applied a pre-determined coding scheme, then achieved consensus ratings. An overwhelming majority of participants affirmed the importance of being able to remain in their current home (94.8%) or community (95.3%) as they age. Top hopes for aging in place centered around health/medical; housing/home services; and independent rural lifestyle. The top worries were related to health/medical; housing/home services, and transportation. Resource awareness was low: 43.3% of all participants were not aware of any available resources. Overwhelmingly, rural residents hope to grow older at home, but may not know how to connect to resources that support this goal. The resource needs we identified are being used to inform community-driven approaches to improve both awareness and availability of community resources in these rural communities.

